# Supplementary material for: Psychometric characteristics and factorial structures of the Defensive Pessimism Questionnaire—Spanish Version (DPQ-SV)
Source: PLoS One. 2020 Apr 17;15(4):e0229695. doi: 10.1371/journal.pone.0229695 (PMC7164620; doi:10.1371/journal.pone.0229695)
Supplement: S1 Table — (DOCX) [file pone.0229695.s001.docx]

**S1Table**. Mardia tests of multivariate skew and kurtosis

|  |  | $\chi^{2}(Skew)$ |  | p-value |  | $Z-value(Kurtosis)$ |  | p-value |
| --- | --- | --- | --- | --- | --- | --- | --- | --- |
| Model 1 |  |  |  |  |  |  |  |  |
|  | One Factor- with item 10 | 1020.68(11.36) |  | <.01 |  | 16.16(193.52) |  | <.01 |
|  |  |  |  |  |  |  |  |  |
| Model 2 |  |  |  |  |  |  |  |  |
|  | One Factor- without item 10 | 752.15(8.37) |  | <.01 |  | 13.59(162.8) |  | <.01 |
|  |  |  |  |  |  |  |  |  |
| Model 3 | Two Factors- with item 10 |  |  |  |  |  |  |  |
|  | Reflectivity-with item 10 | 343.01(3.79) |  | <.01 |  | 7.88(54.65) |  | <.01 |
|  | Negative Expectation | 203.63(2.25) |  | <.01 |  | 5.85(52.74) |  | <.01 |
|  |  |  |  |  |  |  |  |  |
| Model 4 |  |  |  |  |  |  |  |  |
|  | Reflectivity -without item 10 | 175.55 (1.94) |  | <.01 |  | 5.32 (38.83) |  | <.01 |
|  | Negative Expectation | 203.63(2.25) |  | <.01 |  | 5.85(52.74) |  | <.01 |
