# Supplementary figures and images for: Psychometric characteristics and factorial structures of the Defensive Pessimism Questionnaire—Spanish Version (DPQ-SV)
Source: PLoS One. 2020 Apr 17;15(4):e0229695. doi: 10.1371/journal.pone.0229695 (PMC7164620; doi:10.1371/journal.pone.0229695)

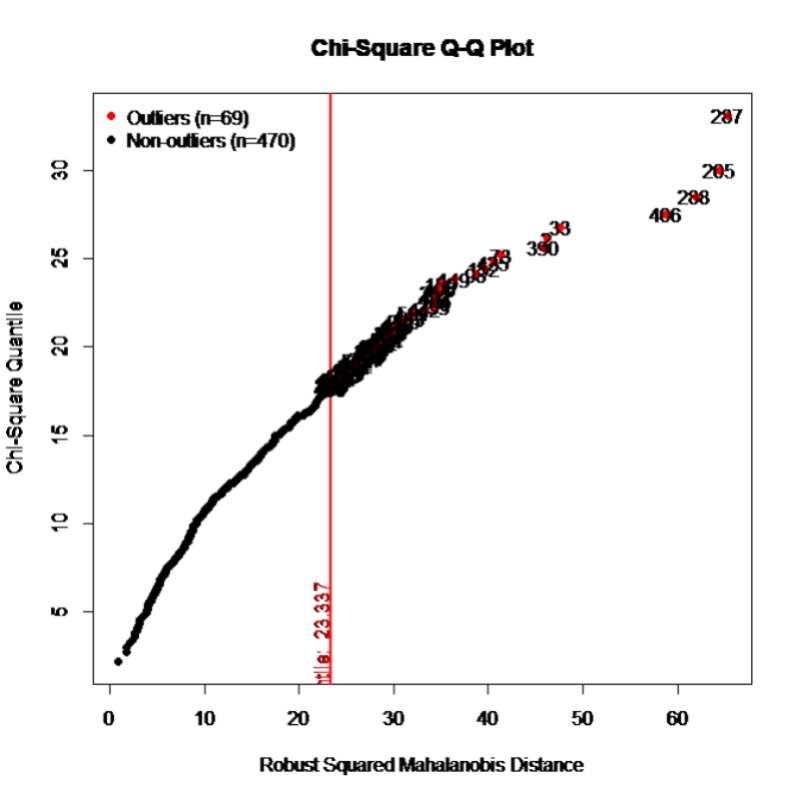

Supplement: S1 Fig — Model 1 (left). (TIF) [file pone.0229695.s002.tif]

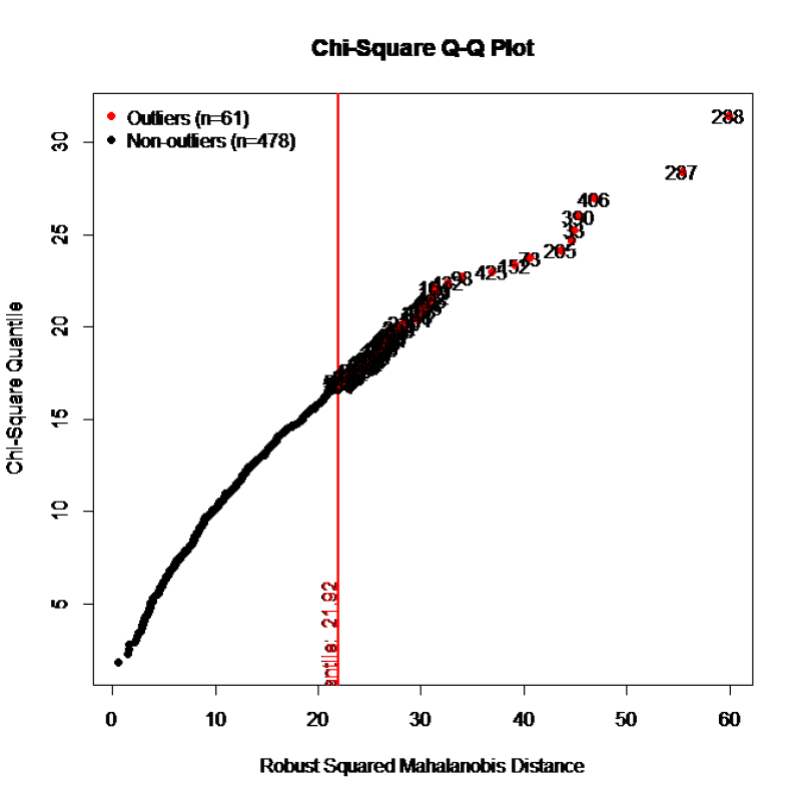

Supplement: S2 Fig — Model 2 (right). (TIF) [file pone.0229695.s003.tif]

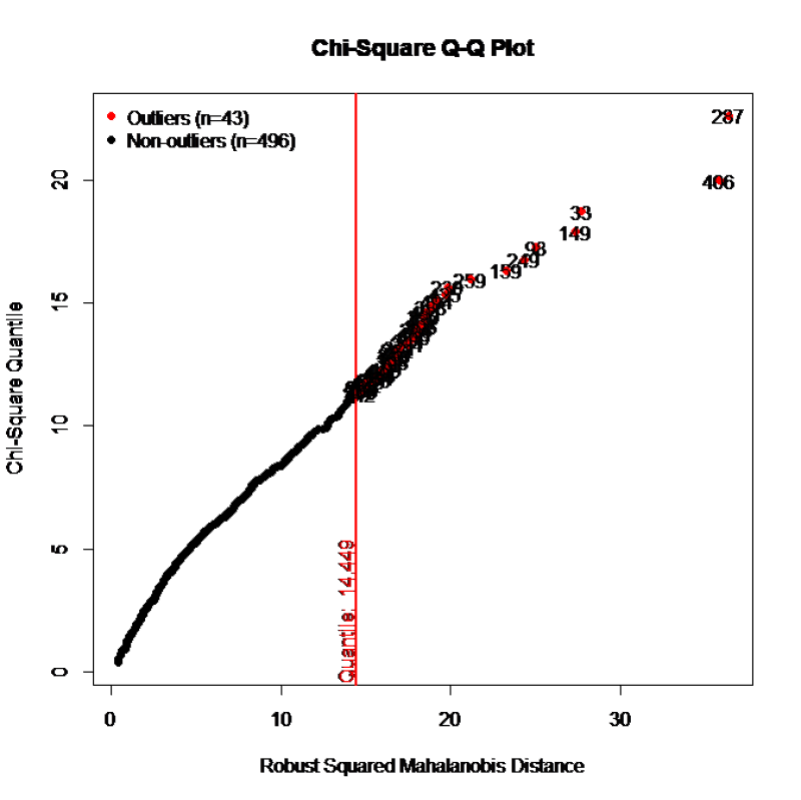

Supplement: S3 Fig — Reflectivity (Model 3). (TIF) [file pone.0229695.s004.tif]

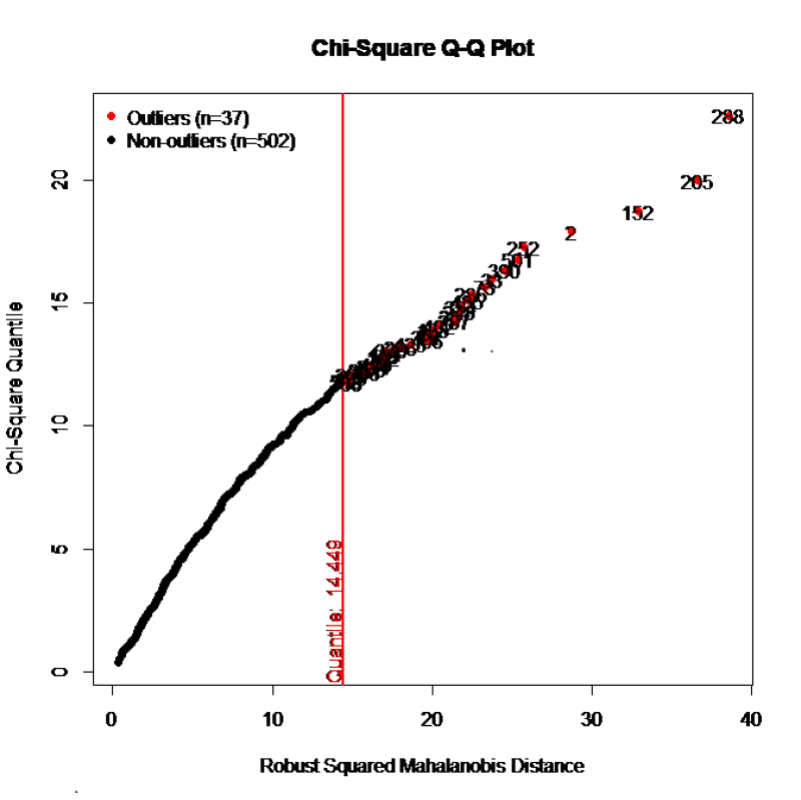

Supplement: S4 Fig — Negative Expectation (Models 3 & 4). (TIF) [file pone.0229695.s005.tif]

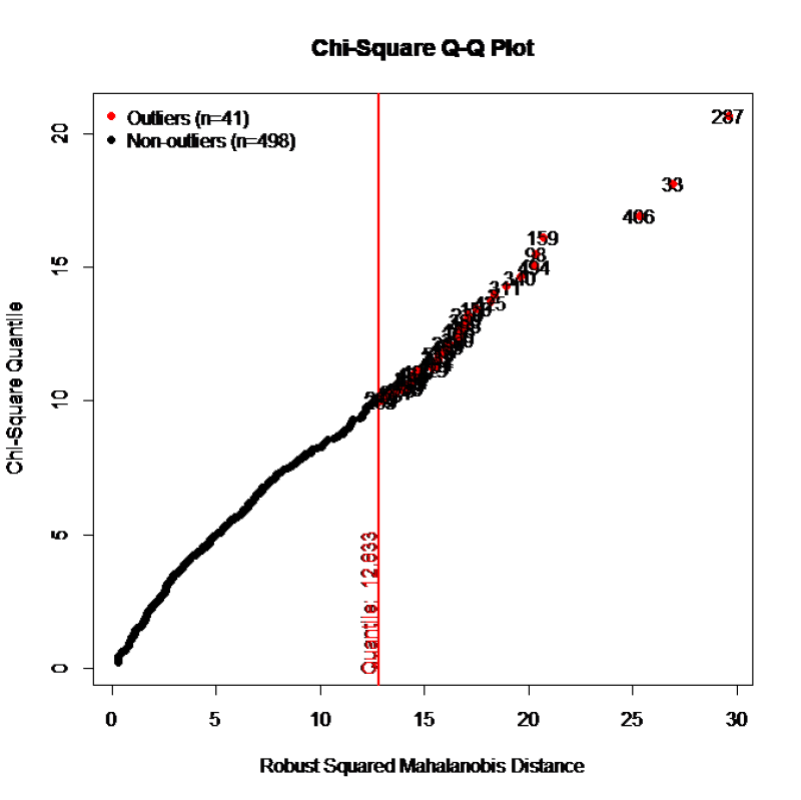

Supplement: S5 Fig — Reflectivity (Model 4). (TIF) [file pone.0229695.s006.tif]
