# Supplementary material for: Psychometric characteristics and factorial structures of the Defensive Pessimism Questionnaire—Spanish Version (DPQ-SV)
Source: PLoS One. 2020 Apr 17;15(4):e0229695. doi: 10.1371/journal.pone.0229695 (PMC7164620; doi:10.1371/journal.pone.0229695)
Supplement: Attachment — Submitted filename: COVER LETTER-PESS_PLOSOne.docx [file pone.0229695.s007.docx]

***PLOSOne***

Dear Editor:

We very much appreciate your new suggestions regarding our paper (***PONE-D-19-28221
Psychometric Characteristics and Factorial Structures of the Defensive Pessimism Questionnaire - Spanish Version (DPQ-SV)***. We have revised the paper accordingly and hope the new version is now acceptable for publication.

This research was supported by grants from from the Spanish Ministry of Science and Innovation (PSI2013-42512- P), and the Regional Government of Andalusia (HUM-566; CTS-278).

This manuscript has not been previously published and has not been submitted elsewhere, however, part of the samples are the same that the ones included in: Ramírez-Maestre**, C.;** Correa, M.; Rivas, T.; López-Martínez, AE.; Serrano-Ibáñez, E.R.; & Esteve, R. (2017). Psychometric Characteristics of the Flourishing Scale-Spanish Version (FS-SV). The factorial structure in two samples: Students and patients with chronic pain. *Personality and Individual Differences, 117,* 30-36*.* [*http://dx.doi.org/10.1016/j.paid.2017.05.035*](http://dx.doi.org/10.1016/j.paid.2017.05.035)

Data can be found in Mendeley: <https://data.mendeley.com/datasets/zvj6cz68sc/draft?a=4f07be08-450a-49e9-bdb6-12fbf002c74c>

The corresponding author (Carmen Ramírez-Maestre) has been authorized by all co-authors to act as an agent on their behalf in all matters pertaining to publication of the manuscript, and the order of names has been agreed by all authors.

**Disclosure statement.** The authors declare no conflicts of interest. None of the authors have any financial relationship with the organization that sponsored the study.

**Funding.** This research was supported by grants from from the Spanish Ministry of Science and Innovation (PSI2013-42512- P), and the Regional Government of Andalusia (HUM-566; CTS-278).

**Informed consent.** All the procedures followed were in accordance with the ethical standards of the committee responsible for human experimentation (institutional and national) and with the 1975 Declaration of Helsinki (revised 2000).

The Ethics Committee of the University of Málaga approved this study (CEUMA 2013-0016-H).

Yours faithfully

Ph. D. Carmen Ramírez –Maestre

e-mail: cramirez@uma.es
